# Representing glycophenotypes: semantic unification of glycobiology resources for disease discovery

**DOI:** 10.1093/database/baz114

**Published:** 2019-11-18

**Authors:** Jean-Philippe F Gourdine, Matthew H Brush, Nicole A Vasilevsky, Kent Shefchek, Sebastian Köhler, Nicolas Matentzoglu, Monica C Munoz-Torres, Julie A McMurry, Xingmin Aaron Zhang, Peter N Robinson, Melissa A Haendel

**Affiliations:** 1 Oregon Clinical & Translational Research Institute, Oregon Health & Science University, Portland, OR 97239, USA; 2 OHSU Library, Oregon Health & Science University Library, Portland, OR 97239, USA; 3 Monarch Initiative, monarchinitiative.org; 4 Linus Pauling Institute, Oregon State University, Corvallis, OR 97331, USA; 5 Charité Centrum für Therapieforschung, Charité-Universitätsmedizin Berlin Corporate Member of Freie Universität Berlin, Humboldt-Universität zu Berlin and Berlin Institute of Health, Berlin 10117, Germany; 6 European Bioinformatics Institute (EMBL-EBI), Wellcome Trust Genome Campus, Cambridge, UK; 7 The Jackson Laboratory for Genomic Medicine, Farmington, CT 06032, USA

## Abstract

While abnormalities related to carbohydrates (glycans) are frequent for patients with rare and undiagnosed diseases as well as in many common diseases, these glycan-related phenotypes (glycophenotypes) are not well represented in knowledge bases (KBs). If glycan-related diseases were more robustly represented and curated with glycophenotypes, these could be used for molecular phenotyping to help to realize the goals of precision medicine. Diagnosis of rare diseases by computational cross-species comparison of genotype–phenotype data has been facilitated by leveraging ontological representations of clinical phenotypes, using Human Phenotype Ontology (HPO), and model organism ontologies such as Mammalian Phenotype Ontology (MP) in the context of the Monarch Initiative. In this article, we discuss the importance and complexity of glycobiology and review the structure of glycan-related content from existing KBs and biological ontologies. We show how semantically structuring knowledge about the annotation of glycophenotypes could enhance disease diagnosis, and propose a solution to integrate glycophenotypes and related diseases into the Unified Phenotype Ontology (uPheno), HPO, Monarch and other KBs. We encourage the community to practice good identifier hygiene for glycans in support of semantic analysis, and clinicians to add glycomics to their diagnostic analyses of rare diseases.

## Introduction

From antiquity to present days, clinicians have described diseases with phenotypic features mostly in a free-text representation—from ancient Egyptians using papyrus (1) to today’s disease descriptions in textbooks, publications or medical records. However, with the advance of bioinformatics methods and standards, phenotypes are increasingly being codified in a computable format using ontologies (2). An ontology provides logical classifications of terms in a specified domain and the relationships between them. It also bears textual and logical definitions, synonyms identifiers and cross-references to other ontologies, databases (DB) and knowledge bases (KB) (3). The Open Biological and Biomedical Ontology (OBO) Foundry has developed standards for logically well-formed and interoperable ontologies respectful of the representations of biological reality (4). These ontologies are often used in KBs and DBs to semantically structure information and allow for computational classification and inferencing across data.

Biomedical phenotype and disease ontologies have been used in precision medicine for ‘deep phenotyping’ (5), which is ‘the precise and comprehensive analysis of phenotypic abnormalities in which the individual components of the phenotype are observed and described’ (6). The Human Phenotype Ontology (HPO) (7) is one of the leading biomedical phenotype ontologies and is used by various European and American national rare disease efforts and clinical databases such as 100,000 Genomes Project (8), ClinGen (9), Orphanet (10) and ClinVar (11). The HPO is a source of computable phenotypic descriptions that can support the differential diagnosis process. For example, a set of HPO-encoded phenotypes from a patient with an undiagnosed disease can be compared with the phenotypes of known diseases using semantic similarity algorithms for disease diagnostics (7, 12–15). The HPO is a part of a reconciliation effort to align the logical representation of phenotypes across species (7), which enables their integration into a common, species-independent resource called the Unified Phenotype Ontology (uPheno) (16). These resources provide the basis of semantic similarity algorithms implemented within variant prioritization tools such as the program Exomiser developed by the Monarch Initiative team (14, 17), which uses a protein-interaction network approach to help prioritize variants based on interaction partners (18–20). The Monarch Initiative (monarchinitiative.org) provides ontology-based tools for clinical and translational research applications (12–14). The Monarch platform uses the Mondo Disease Ontology that provides a harmonized and computable foundation for associating phenotypes to diseases (21, 22). Mondo integrates the existing sources of disease definitions, including the Disease Ontology (23), the National Cancer Institute Thesaurus (NCIt) (24), the Online Mendelian Inheritance in Man (OMIM) (25), Systematized Nomenclature of Medicine–Clinical Terms (SNOMED CT) (26), International Classification of Diseases (27), International Classification of Diseases for Oncology (28), OncoTree (29), MedGen (30) and numerous others into a single, coherent merged ontology. Mondo is co-developed with the HPO, to ensure comprehensive representation of diseases and phenotypes.

Use of semantic deep phenotyping approaches has been particularly valuable in cases, where a strictly sequence-based analysis has been insufficient to lead to a diagnosis. This is often the case with patients admitted to national and regional undiagnosed clinics, such as the National Institutes of Health (NIH), Undiagnosed Diseases Program (UDP) and Network (UDN), where only 28% of UDN patients have been diagnosed to date (31). One of the most interesting characteristics of patients in these programs is the high incidence of glycan-related molecular defects, which we refer to here as ‘glycophenotypes’. These include observable abnormalities in the structure, abundance, distribution and activity of glycans, as found in their free or conjugated forms. For example, Gall *et al.* (32) reported that 50% of patients admitted to the UDP had abnormal glycophenotypes, whether the causal genes were related to glycobiology or not (33). While diseases related to glycobiology have been well-studied (34–36), the integration of glycomics data and glycophenotypes into biological KBs lags behind what we see for genomic, proteomic and metabolomic data (key biological entity types like genes, diseases, pathways, etc.); hence, ‘the need of informatics in glycobiology’ as Campbell *et al.* state: *‘Databases that provide authoritative information about glycan and glycoconjugate structures, and well-defined standards that allow this information to be exchanged, are required as a foundation for computational tools that give insight into the biological functions and consequences of glycosylation* (37)’.

**Figure 1:**
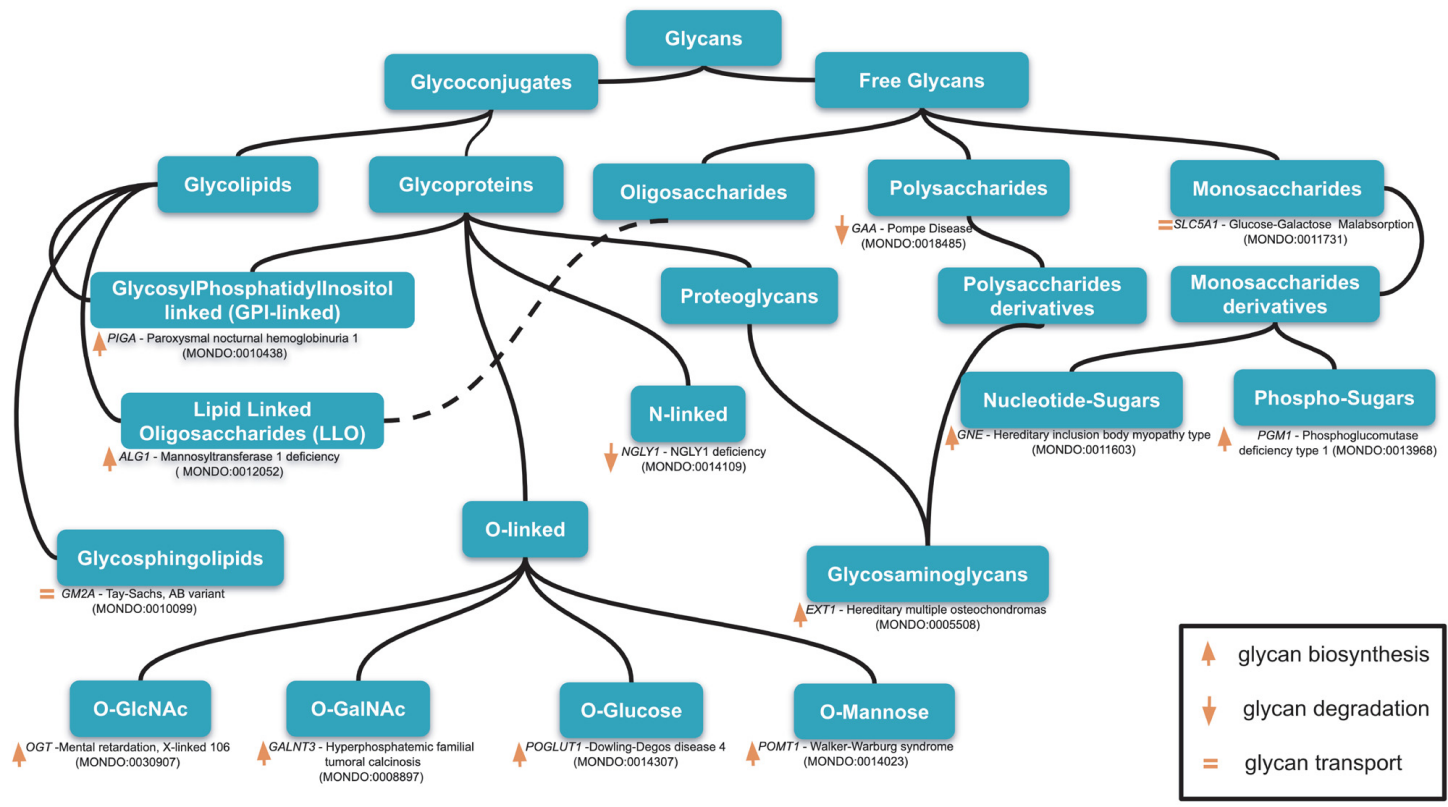
An example of classification of relevant glycans involved in human diseases based on the essential of glycobiology and CHEBI ontology. Glycans can be free or conjugated to macromolecules (protein, lipids). Free glycans can be monosaccharides (*n* = 1), oligosaccharides (2 < *n* < 10) or polysaccharides (*n* > 10), and their derivatives (e.g. acetylated, sulfated) 13 exemplary diseases names along with the mutated genes and MONDO ID (in parentheses) are indicated for 13 classes of glycans. Up, down orange arrows and orange equal signs indicate, respectively, the involvement of the gene products in glycan biosynthesis, degradation or transport. Based on our disease curation, there are 176 glycan-related diseases (CDG and diseases in which glycophenotypes are detectable, see online supplementary material for Table S1).

Despite the diagnostic and informatics success of HPO, glycophenotypes are underrepresented in this resource and, thus, limit their value in differential diagnosis. For instance, patients with fucosidosis can have at least five glycophenotypes such as decrease of fucosidase activity, urinary glycosaminoglycan excretion, oligosacchariduria, increase of urinary glycopeptides and accumulation of glycolipids expressing blood group antigens in the liver (38, 39), but only two of these glycophenotypes are in HPO (40, 41). In addition to phenotypes related to glycan-binding protein (GBP) staining, there can be potentially at least six glycophenotypes per glycan-related diseases, hence, 1056 possible HPO glycophenotypes terms for the 176 glycan related diseases (congenital disorder of glycosylation (CDG) and diseases where glycophenotypes are detectable, see online supplementary material for Table S1). Nevertheless, there are only 126 HPO terms related to glycobiology as of time of writing, which are either subclasses of abnormal glucose homeostasis (38/126), abnormal glycosylation (46/126 whether glycolipid metabolism or protein glycosylation), abnormal glycosyltransferases activity (4/126) and abnormal free glycans (38/126), (see online supplementary material for Table S2). In addition, these existing HPO glycophenotypes occupy only 5/20 categories of glycans indicated in Figure 1 (glycosyltransferases and GBP phenotypes excluded). Finally, within Monarch platform, the term ‘abnormal glycosylation’ (HP:0012345) is associated with 31 diseases (42) and the term ‘glycan’ returns only 17 phenotypes (43), yet according to Freeze *et al.* (34) and Ferreira (44), 130–134 CDG exist (non-including other glycan-related diseases such as diseases related to GBP). Furthermore, Xia *et al.* (36) reported 54 different urinary glycophenotypes for 10 glycan-related diseases.

Here, we provide a short overview of glycobiology, its importance in health and disease and a discussion of some of the technological and informatics challenges that face the use of these data for disease discovery research and diagnostics. We demonstrate the utility in adding glycophenotypes to disease diagnostic pipelines. Finally, we present the results of surveying a selection of ontologies and KBs based on an updated list of glycoscience-related informatics resources from Campbell *et al.* (37).

## Glycans: A Galactic Odyssey

### Structures and classification

Glycans, also referred to as carbohydrates and sugars (45), are a fundamental class of biomolecules with the general chemical formula C*_n_*H_2*n*_O*_n_*. They are among the oldest organic molecules found in the Milky Way, and one of the simplest glycans, glycoladehyde, was even discovered on the molecular cloud Sagittarius B2 (46). Glycans, along with amines, may have enriched our solar system to influence life on Earth (47), possibly during meteorite collisions on planet Earth, which led to the formation of more organic molecules (48).

Glycans can be found in bacteria, archaea, eukaryotes and most viruses (49). They are the most abundant biomolecules on Earth with plant-synthesized cellulose (50). In eukaryotic cells, glycans can be found in free forms (monosaccharides, oligosaccharides and polysaccharides) or as bioconjugates, covalently attached to the other major classes of biomolecules such as nucleic acids (sugar-nucleotides), proteins (glycoproteins with *N*-linked glycans (N-glycans) and O-linked-glycans (O-glycans), glycosylphosphatidylinositol-anchored, proteoglycans) and lipids (lipid-linked oligosaccharides, glycosphingolipids, glycosylphosphatidylinositol-anchored) (Figure 1).

Glycans can be *N*-acetylated (e.g. *N*-acetylglucosamine or GlcNAc), phosphorylated (e.g. glucose-6-phosphate), sulfated (e.g. heparan sulfate [HS]), etc. The collection of all glycans in an organism, the glycome, displays an extreme diversity of structures, amounting to up to 10^4^ times more molecules than those found in the proteome (51). The molecular weight of glycans can range from 60 with glycoladehyde (46) to more than 2 × 10^6^ Daltons (Da), with glycan polymers such as hyaluronan (52) making them only partially accessible to metabolomics studies, as metabolomics focuses on the study of molecules below 1,500 Da (53). Glycans, although often regarded at the periphery of metabolomics, proteomics and lipidomics, can play crucial roles in cell biology.

## Glycan Roles in Human Biology

Given their ancient evolutionary history, diversity and abundance, it is not surprising that glycans play a pivotal role in human biology. Glycans play many roles that range from structural, modulatory to recognition (49) (Table 1). In terms of structural role, glycans can be a physical barrier, assist protein folding and serve as energy storage. The physical barrier or glycocalyx is a protective coat made of glycoaminoglycans (e.g. HS), glycoproteins (including GPI-anchored) and glycolipids located at the cell surface of eukaryotic cells (54). Glycans at the cell surface and on circulating serum proteins can also provide a shield against proteases and against attachment to certain pathogens (45). Glycans can help protein folding in the endoplasmic reticulum by stabilizing and promoting interaction with GBP (lectins) involved in protein folding such as calnexin and calreticulin (55). Glycans can serve as a structural energy storage with glycogen made of polymer of 55 000 molecules of glucose (56).

**Table 1:** Glycan roles, exemplary HPO terms and glycophenotypes associated with six genetic diseases

Glycan roles		Glycan-related group and pathways	Mutated gene	Disease (Mondo identifier)	Abnormal phenotypes associated with disease	
					Abnormal glycophenotypes	Examplary anatomical, infectious and behavioral phenotypes
Structural	Physical barrier	Glycosaminoglycans (HS polymerization)	*EXT1*	Hereditary multiple osteochondromas (MONDO:0005508)	Decreased circulating HS level (HP:0410343)	Abnormality of the humerus (HP:003063) Multiple exostoses (HP:0002762)
	Protein folding	O-glycans synthesis Protein folding	*B3GLCT*	Peters Plus syndrome (MONDO:0009856)	Shortened O-fucosylated glycan on properdin (HP:0410344)	Anterior chamber synechiae (HP:0007833) Brachycephaly (HP:0000248)
	Energy storage	Polysaccharide (glycogen degradation)	*GAA*	Pompe Disease (MONDO:0009290)	Increase of urinary polyhexose glycans (HP:0410345)	Cardiomegaly (HP:0001640) Cognitive impairment (HP:0100543)
Modulatory	Signaling	O-Glycans synthesis (O-Fucosylation) Notch signaling	*LFNG*	Spondylocostal dysostosis 3 (MONDO:0012349)	Decreased glycosyltransferase O-fucosylpeptide 3-β-*N*-acetylglucosaminyltransferase activity (HP:0410349)	Scoliosis (HP:0002650) Slender finger (HP:0001238)
Recognition	Intrinsic	O-glycans synthesis (O-mannosylation) laminin-dystroglycan binding	*POMT1*	Muscular dystrophy-dystroglycanopathy type A1 (MONDO:0014023)	Hypoglycosylation of alpha-dystroglycan (HP:0030046)	Cataract (HP:0000518) Intellectual disability, severe (HP:0010864)
	Extrinsic	GBP to pathogen Toll-like receptor signaling Creation of C4 and C2 activators	*MBL2*	Mannose-binding lectin (MBL) deficiency (MONDO:0013714)	Decreased mannose-binding protein level (HP:0032305)	Recurrent *Klebsiella* infections (HP:0002742) Failure to thrive (HP:0001508)

The modulatory role of many signaling proteins depends on their own glycosylation, the glycosylation or glycan binding activity of their counter receptors. For instance, human chorionic gonadotropin’s signal transduction depends on its *N*-linked glycans (57). Similarly, glycans on cell surface proteins are required for the signaling of GBP. For instance, galectin-1 and galectin-8 will signal phosphatidylserine exposure on neutrophils through interaction with polylactosamine containing counter receptors (58, 59). Finally, some receptors can be regulated by signaling glycans such as GM3 glycolipid on the epidermal growth factor receptor (EGFR) (60).

Glycans are involved in intrinsic and extrinsic recognition (45, 49). From the initiation of spermatozoid attachment on sialyl-Lewis(x) on the egg (61) to cell death with glycosylation of Fas/TRAIL death receptor (62), glycans play a role in cell recognition and in the cellular social life, through the interaction of glycan–protein or glycan–glycan interaction. For instance, many antigens are glycan-based, such as the ABO blood group (63). Stem cells growth and differentiation depend on O-fucosylation on Notch (64), and HS is a key regulator of embryonic fate (65). In fact, stem cells’ glycosylation profiles indicate the stage of pluripotency, especially fucosylated glycans (66), and are used for their isolation through specific sets of lectins (67). In cancer biology, glycans are used as markers for many types of cancers (68) and are involved in resistance to cancer in naked mole rats with high molecular weight hyaluronan (69). Glycans are also involved in parasitic infections, during the attachment phase, whether zoonotic (e.g. schistosome (70)), microbial (e.g. *Escherichia coli* 086, bearing blood group antigen (71)) or viral (e.g. influenza virus H1N1, where H stands for hemagglutinin and N for neuraminidase, respectively, a lectin and a glyco-enzyme (72)).

## Glycans in Human Diseases

Alterations in glycan function, such as genetic perturbation in synthesis (involving glycosyltransferases, chaperone of glycosyltransferases, transporter, etc.), degradation or their attachment through GBP, can contribute to the pathophysiology of various diseases. For instance, mutations in the glycosyltransferase *EXT1* can lead to the formation of abnormally short HS molecules which accumulate in the Golgi apparatus, and cause abnormal cytoskeleton formation (73) and increase of bone morphogenetic proteins that lead to osteochondromas (74). Glycans also play a role in molecular recognition in innate and acquired immunity. Human milk oligosaccharides contribute to a healthy infant gut microbiome by preventing bacteria and viruses from binding to the intestinal mucosa (75). Bacterial lipopolysaccharide can stimulate innate immune responses (76). Glycosylation of immunoglobulins (Ig) can contribute to many autoimmune diseases such as IgA nephropathy (77). In this disorder, abnormal hypoglycosylated IgA1 displays the glycan epitope (GalNAc) which is recognized as non-self by specific antibodies, forming IgA-immune complexes that are deposited in the renal mesangium and cause glomerular injury (78). In this example, abnormally hypoglycosylated IgA1 is a glycophenotype associated with IgA nephropathy.

**Figure 2:**
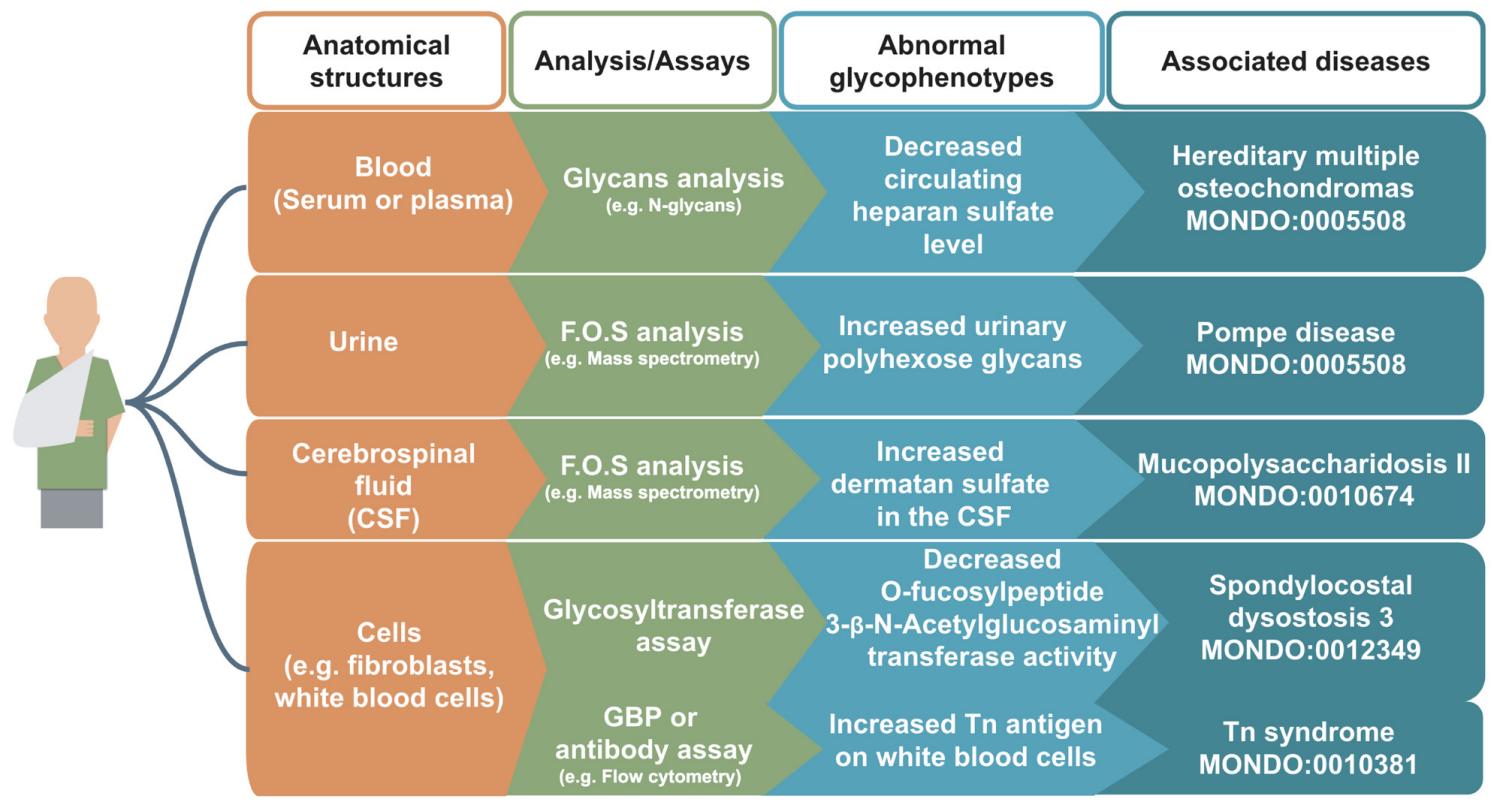
Examples of glycophenotypes that can be captured from various laboratory techniques From a patient’s anatomical structures (indicated in orange boxes, e.g. blood), glycans such as free oligosaccharides (F.O.S.) and glycan-related molecules can be analyzed by standard glycomics assays (indicated in green boxes, e.g. GBP or antibody assay). Patients’ glycophenotypes indicated in the blue boxes can be captured from publications: decreased circulating HS level (82), increased urinary polyhexose glycans (36), increased dermatan sulfate in the CSF (83), decreased O-Fucosylpeptide 3-beta-*N*-acetylglucosaminyltransferase activity (84) and increased Tn antigen in white blood cells (85). In our preliminary work, we have logically defined design patterns (86) that would generate hundreds of classes, but they are not yet fully integrated in the HPO.

Exemplary glycophenotypes measured on biomolecules from cells, tissues and bodily fluids are indicated in Figure 2 and Table 1. For example, assays are performed to quantify and characterize free glycans, glycopeptides, glycoproteins and glycosyltransferase activities in body fluids (urine, blood or serum and cerebrospinal fluid), or in cells, such as fibroblasts. Standard glycomics assays include protein analysis via mass spectrometry, glycosyltransferase activity and glycan binding assays. Glycophenotypes can be described in a structured way as abnormalities of the biomolecule in a given anatomical location, such as abnormal glycopeptide level in the blood.

Abnormalities in the structure, abundance, location and biological activity of glycans have been identified in over one hundred genetic disorders, including diseases with abnormalities of glycan degradation, congenital disorders of glycosylation (CDG) and deglycosylation (79). Disorders related to glycosylation often present a multitude of molecular glycophenotypes.

Abnormal glycophenotypes are present in many genetic diseases related to glycobiology as indicated in Table 1, but they have also been described in the ‘fringes’ of our current knowledge of glycan-associated genes (80). For instance, dysfunction in the DNA excision repair enzyme encoded by *ERCC6* (excision repair 6, chromatin remodeling factor) can lead to abnormal fucosylated glycans in the urine, which is a marker for Cockayne syndrome type 2 (81).

## Glycophenotyping to Improve Human Disease Diagnosis: Fucosidosis

**Figure 3:**
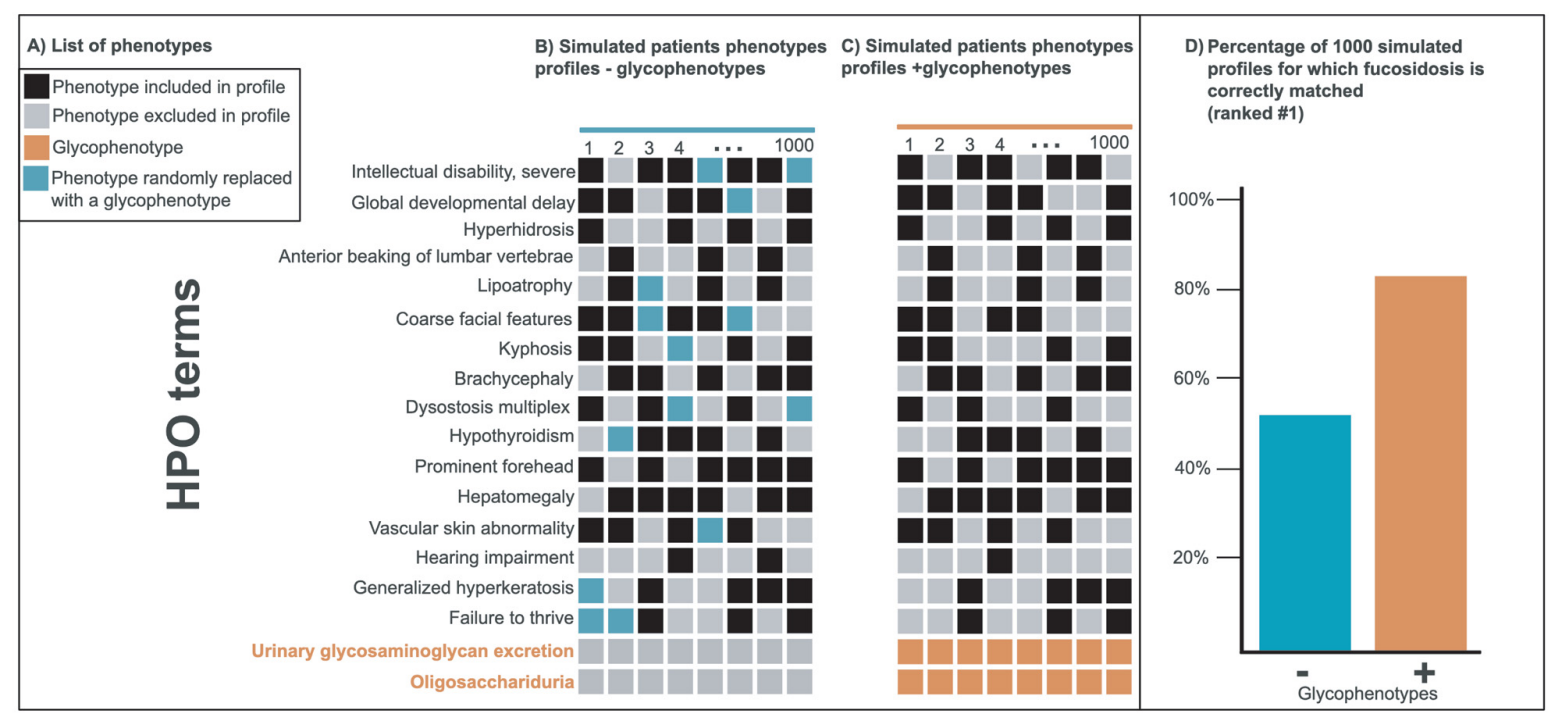
Improvement of disease diagnostic with molecular glycophenotypes for fucosidosis. Panel A lists 18 phenotypes frequently associated with fucosidosis. The columns in Panels B and C illustrate simulated patients phenotypes profiles composed of a random selection of 10 of these 16 phenotypes. The profiles in Panel C include glycophenotypes (bottom in orange), whereas those in Panel B do not. Panel D shows that when these two groups of 1000 simulated profiles are compared for their diagnostic utility, the profiles that contain glycophenotypes (C) significantly outperform those that do not (B) (Fisher exact *P*-value = 8.5e-47). Moreover, more specific glycophenotypes are more diagnostically useful than more general ones. This underscores the importance of harmonizing glycophenotypes across data resources as well as collecting them from patients.

Within the Monarch Initiative platform, whereas hepatomegaly is a common phenotype for 494 diseases (87), oligosacchariduria is a distinctive glycophenotype for nine diseases (40). As unique glycophenotypes can be markers for specific diseases, we hypothesized that expanding the representation of glycophenotypes in the HPO and their use in disease annotations could improve phenotype-based comparisons. As a proof of concept, we performed an analysis with phenotypes associated with the disease fucosidosis (MONDO:0009254). We created 1000 ‘simulated’ patients’ profiles by randomly sampling 10 out the 16 most frequently associated phenotypes with fucosidosis within Monarch Initiative platform (Figure 3A and B). In a second set, we randomly replaced two phenotypes (blue squares, Figure 3B) with two glycophenotypes known to be associated with fucosidosis for each profile (orange squares, Figure 3C). Both sets were compared using the PhenoDigm algorithm (17), which given a list of phenotypes, ranks candidate diseases based on phenotypic similarity. As shown in Figure 3D, the addition of glycophenotypes led to a higher ranking for fucosidosis (rank #1: 82% with glycophenotypes versus 53% without the two glycophenotypes with a Fisher exact *P*-value = 8.5e-47). The workflow is available in a Jupyter notebook (88) at http://bit.ly/glycop_owlsim_analysis.

This demonstrates that the addition of glycophenotypes improves the ranking of relevant glyco-related diseases for candidate diagnoses. Thus, we believe that broadening the representation of molecular phenotypes in phenotype ontologies could help refine rare disease diagnoses.

## Landscape Review of Glycobiology and Glyco-Disease Resources: A Need for Improved Glycan Representation and Standards

To the best leverage abnormal glycophenotype data in bioinformatics analyses, as with metabolomics data for the prioritization of pathological variants for whole genome/exome sequencing (WGS/WES) (89), we need comprehensive, standardized and connected representations of glycan terminology. This requirement includes interoperable representation of glycophenotypes in biomedical ontologies, KBs and DBs. While the nomenclature for glycan-related diseases is well established, the nomenclature for glycans can be complex. We face several hurdles to increase the standardized representation of glycophenotypes:

(1) Lack of a unified standard terminology and identifiers for glycans and glycan related entities (e.g. effort such as GlycoCT (90) and the Symbol Nomenclature for Graphical Representations of Glycans (SNFG) (91), respectively, for encoding scheme and pictorial representation of glycans, but they are not mandatory in all journals).
(2) Technical/experimental barriers for interrogating glycophenotypes (standard mass spectrometry cannot differentiate monosaccharide epimers and define linkage between monosaccharides).
(3) Lack of coverage of glycan-related concepts in ontologies (for instance, the Chemical Entities of Biological Interest (CHEBI) ontology (92) contains 264 entries, while more than 100 000 unique cross-species and synthetic glycan structures exist in the glycan repository GlyTouCan (93) and 15 000 unique glycan structures have been estimated for humans (94)).
(4) Lack of associations of glycans and glycan abnormalities with diseases and phenotypes in existing databases such as Monarch (12).
(5) Lack of interoperability across DBs/KBs containing glycan-related data/knowledge (for instance, the semantic barrier between scientific disciplines related to glycobiology as the same molecule can be referred to differently in different subdisciplines of immunology, hematology, biochemistry: CD173, blood group O, H-type 2 antigen (95) or Fuc(α1-2)Gal(β1-4) GlcNAc (96)).
(6) Lack of queryable data store based on glycophenotypes, levels, location, subjects, assays, abnormalities, evaluand, gene, etc. (e.g. an increase/decrease or presence/absence of a particular glycophenotype in a given bodily fluid in a given genetic disease, see Figure 2).
(7) Lack of human and machine-readable formats for diseases, glycans and phenotypes (e.g. International Union of Pure and Applied Chemistry, IUPAC (97)).
(8) Lack of glycophenotype algorithms for disease comparison based on structured rules (e.g. increase of Tn antigen associated with mutation on *C1GALT1C1* for cancer (98)), logical structure and relationships between entities (e.g. fuzzy phenotype search (13)).

Hence, there is a need for a standardized vocabulary and identifiers, best practices to facilitate the curation of glycophenotypes related to genetic diseases, especially as the human glycome project aims to define the structures and functions of human glycans which have started (99).

Regardless of this complexity, many glycobiology-related KBs exist with differences in specificity (glycan-related enzymes, diseases, molecules, etc.) that could be used for disease and glycan comparisons in a human readable and computable manner. In Table 2, we provide a review of existing resources and identified gaps and opportunities for additional development in both the HPO and cross-species phenotype ontologies and glyco-KBs and DBs. We focused particularly on diseases and/or glycans by highlighting features, applications, uses and challenges in order to provide potential resources for representing glycophenotypes in a compatible way with the HPO, and applying them toward phenotype-based patient diagnosis and disease-gene discovery.

**Table 2:** Description of the KB and Ontologies Overview of relevant knowledge bases and ontologies based on their contents and glycan related data (glycophenotypes, glycan related diseases, genes, GBP, etc.)

Resources (names and links)	Domains	Descriptions
**Knowledge base**		
CAZY (100)http://www.cazy.org/	Glyco-genes	CAZY has curated data from publications on carbohydrate-active enzymes responsible for the synthesis and breakdown of glycoconjugates, oligosaccharides and polysaccharides. It provides classification of these glyco-enzymes based on their activities (glycoside hydrolases, glycosyltransferases, polysaccharide lyases, carbohydrate esterases and auxiliary activities) and glycan-related genes browser in different species
CFG (101)http://www.functionalglycomics.org/	Glyco-genes GBP glycans diseases	The CFG has generated and collected publicly available data on GBPs (glycan array), glycan profiles in cells and tissue, phenotypic analyses of transgenic mouse lines with knockout glycan related genes (histology, immunology, hematology and metabolism/behavior)
GlyConnect (102)https://glyconnect.expasy.org	Glyco-genes GBP Glycans Diseases	GlyConnect integrates of information about protein glycosylation for different species based on taxonomy, protein, tissue, composition disease, glycosylation sites, peptides and references
GlycoSciences (103)http://glycosciences.de/	Glycans diseases	GlycoSciences provides experimental information for glycans such as structure, composition, motifs, biophysical experiments on glycans and curation of comprehensive repository of cluster of differentiation (CD) antigens
GlyTouCan (93)https://glytoucan.org/	Glycans	Glytoucan is a free glycan repository that provides unique accession numbers to any glycan independently of experimental information (*n* = 110 668). GlyTouCan has made efforts to bridge gaps between experimental glycans and native glycans by creating identifiers from the mass spectrometry data and encourages glycobiologists to use them in their publications
JCGGDB (104)https://jcggdb.jp/database_en.html	Glyco-genes GBP glycans diseases	JCGGDB is an integrative database for glycan information and diseases using different resources. It has compiled information related to glycan-related genes or GlycoGene (enzymes, transporter, etc.) and glycan diseases (e.g. CDG-Ia), pathosis, links to other KBs associated gene descriptions (e.g. PMM2) and a genetic glyco-diseases ontology that provides a hierarchical classification of the diseases
KEGG (105)https://www.genome.jp/kegg/glycan/	Glyco-genes GBP glycans diseases	KEGG is a KB that includes a module dedicated to glycobiology (KEGG-glycan) in which glycan identifiers, glycan pathways, genes, and links to other glycan databases. It allows for the search of glycan terms (abbreviation and synonyms) and gives composition, identifiers, reaction, pathways, etc.
Monarch (13)monarchinitiative.org	Glyco-genes GBP glycans diseases	Monarch initiative is a platform that provides analytic tools and web services for cross-species comparison of genotype–phenotype associations, disease modeling and precision medicine using semantically integrated data
OMIM (25)https://omim.org/	Glyco-genes GBP diseases	OMIM is a resource containing information about human genes and genetic disorders. It provides information on genetic diseases and associated phenotypes, including disease names and synonyms, unique, phenotype-gene relationships, descriptions of diseases (diagnosis, pathogenesis), clinical and biochemical features, genetic information as well as animal models
PubChem (106)https://pubchem.ncbi.nlm.nih.gov/	Glyco-genes GBP glycans diseases	Pubchem is an open KB from the NIH for chemical structures, identifiers, chemical and physical properties (biological activities, patents, health, safety, toxicity data, etc.). Data can be queried online or downloaded (JSON, XML, ASN.1 files)
Reactome (107)https://www.reactome.org/	Glyco-genes GBP glycans diseases	Reactome is an open-source and peer-reviewed pathway KB that allows search based on biological terms. Reactome has a repertoire of diseases of glycosylation (related to GAG, *N*-glycans synthesis, O-glycans synthesis and precursors of glycosylation)
UniLectin (108)https://www.unilectin.eu/	GBP glycans	UniLectin is an interactive KB that classifies and curates GBP (or lectin) and their ligands
**Ontologies**		
CHEBI (92)https://www.ebi.ac.uk/chebi/	Glycans	CHEBI is a dictionary for small molecules developed by the European Bioinformatics Institute using sources from KEGG and developed with an ontology framework. It provides an identifier, name, annotation rating, structure, molecular formula, charge, average mass, ontology, etc.
GO (109)http://geneontology.org/	Glyco-genes GBP glycans	GO consortium is an initiative for the computational representation of genes and their biological functions at the molecular, cellular and histological levels. It provides gene annotations, ontology, mapping and tools such as gene enrichment analysis
NCIt (24)https://ncit.nci.nih.gov/ncitbrowser/	Glyco-genes GBP glycans	NCIt is a thesaurus from the National Cancer Institute Enterprise Vocabulary Services. It provides concepts, terminology, therapies related to cancer and related biomedical topics

We reviewed a selection of widely used KBs and ontologies with glycan-related content that will help to jump the hurdles we mentioned above. Based on these criteria, we selected relevant KBs/services that could be used to support ontological glycophenotype representations for phenotype-based diagnosis and disease-gene discovery as indicated in Table 2 with CAZY (100), Consortium for Functional Glycomics (CFG) (101), GlyConnect (102), GlycoSciences (103), GlyTouCan (93), Japan Consortium for Glycobiology and Glycotechnology DataBase (JCGGDB) (104), The Kyoto Encyclopedia of Genes and Genomes (KEGG) glycan (105), Monarch (13), OMIM (25), Pubchem (106), Reactome (107), UniLectin (108), CHEBI (92), gene ontology (GO) (109) and NCIt (24).

**Figure 4:**
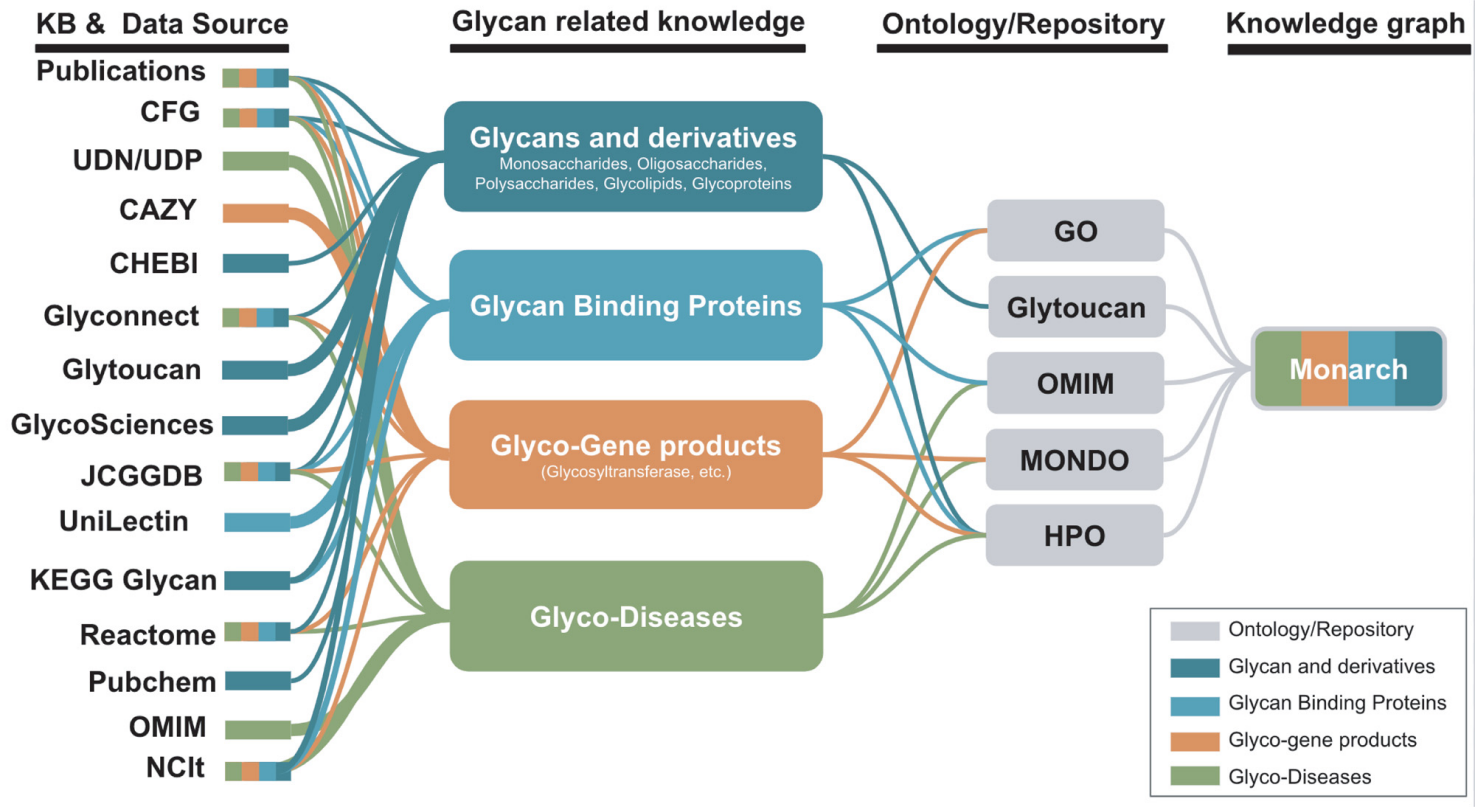
Potential KBs and data sources for the improvement of glycophenotypes representation for HPO and Monarch glycophenotypes related to diseases indicated in publications and KBs could be used to enhance glycan-related knowledge in Monarch.

Following this scope of disease-glycophenotype association, we reviewed key features/applications, use and potential challenges (e.g. using community standards, providing glycan identifiers, links to other resources, etc.) as indicated in Table 3. The majority of the KBs/DBs possess human and machine-readable formats (14/15) and standardized terminologies (15/15) and ontologies (10/15). While all of them have a queryable data store, only two of them possess a phenotype algorithm (Reactome and Monarch) and one has both raw and curated data (CFG). About 8/15 of them have more than 1000 glycans-related terms. Therefore, we propose to build a modular molecular glycophenotype branch that could be integrated into the HPO and other phenotype ontologies as shown in Figure 4.

## A Comprehensive Semantic Representation of Glycophenotypes

### Integration of glycophenotypes in the HPO and the uPheno framework—prototype of a MGPO

The initial high-level classification of molecular glycophenotypes is now available in HPO, for instance ‘Abnormal protein glycosylation’ (HP:0012346) or ‘Abnormality of glycolipid metabolism’ (HP:0010969). Future efforts will include the integration of subclasses of glycophenotypes in HPO using the resources described above as well as glycan-related terms from clinical measures from the Logical Observation Identifiers Names and Codes (LOINC) within the context of the LOINC2HPO project (110). As this work matures, it will be necessary to create rich educational materials and in-line help for glycobiology curators.

Future effort will also include a developed version of the molecular glycophenotype ontology (MGPO) (86). MGPO phenotypes are logically defined according to the patterns defined by the Unified Phenotype Ontology (uPheno) framework wherever possible (16). Complex patterns specific to glycophenotypes (beyond the scope of uPheno) extend existing uPheno patterns. MGPO prototype includes the following primary characteristics (glycan levels, composition, length, occupancy and binding) and secondary dimensions (glycan type, attachment status, location, residue type and residue position) (https://github.com/monarch-initiative/glyco-phenotype-ontology) (86). The MGPO prototype aimed to inform a more comprehensive ontological representation of glycophenotypes. Considering the biochemical diversity of monosaccharides and possible linkages (e.g. chirality of the molecules, anomeric carbon and 2*^k^* stereoisomers, where *k* is the number of carbon atoms (111)), the diversity of glycan chains quickly becomes exponential. To enable this level of expansion while retaining robust and consistent logical structure, we will use ‘Dead Simple Ontology Design Patterns’ (DOSDP) (112). Use of DOSDPs ensures the interoperability of glycophenotype terms with those from other phenotype ontologies and its future integration into uPheno, currently being developed by the Monarch Initiative and collaborators from the Phenotype Ontology Reconciliation Effort (113).

**Table 3:** Review of KB and Ontologies We reviewed relevant knowledge bases and ontologies based on criteria such as presence of human-machine readable, phenotype algorithms, numbers of glycan related terms, etc. Some KBs are richer than other, nevertheless, none of them cover all the criteria

Resources	Human and machine-readable formats	Queryable data store	Phenotype algorithms	Standardized terminologies and ontologies	Type of data	Glycans-related terms (glycan, sugar, carbohydrate, glycoproteins, glycolipid, glycosyltransferase and lectin)
CAZY	No	Yes	No	Many	Curated	333
CFG	No	Yes	No	Many	Raw & curated	>1000
Glyconnect	Yes	Yes	No	Many	Curated	>1000
GlycoSciences	Yes	Yes	No	Many	Curated	>1000
Glytoucan	Yes	Yes	No	Many	Curated	>1000
JCGGDB	Yes	Yes	No	Many	Curated	>1000
KEGG glycan	Yes	Yes	No	Many	Curated	>1000
Monarch	Yes	Yes	Yes	Many	Curated	54
OMIM	Yes	Yes	No	Many	Curated	227
Pubchem	Yes	Yes	No	Many	Curated	252
Reactome	Yes	Yes	Yes	Many	Curated	352
UniLectin	No	Yes	No	Many	Curated	50
CHEBI	Yes	Yes	No	Many	Curated	>1000
GO	Yes	Yes	No	Many	Curated	>1000
NCIt	Yes	Yes	No	Many	Curated	364

**Figure 5:**
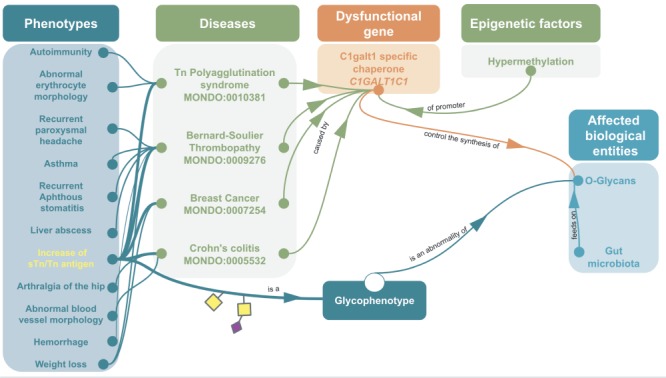
Example of omics integration with ontologies related to glycans: graph representation of the impact of a dysfunctional C1GALT1C1 on health C1GALT1C1 encodes Cosmc, a molecular chaperone for a glycosyltransferase that initiates O-GalNac glycans synthesis (T-synthase) (127). Dysfunctional Cosmc can lead to an improper T-synthase folding, thus abnormal O-glycans with the abnormal glycophenotype: increase of sTn/Tn antigen (SNFG symbols, respectively, yellow square for Tn and a purple diamond/yellow square for sTn) (128). Dysfunctional Cosmc (129) can be due to mutations or epigenetic factors, for instance the hypermethylation of C1GALT1C1’s promoter can lead to increase of sTn/Tn antigen. These two glycophenotypes are also common in many cancers (130). Mouse models have shown that C1GALT1C1 mutation can lead to abnormal O-glycans on platelets, generating bleeding disorders similar to Bernard-Soulier syndrome (MONDO:0009276) (131) Inflammatory bowel disease similar to Crohn’s Colitis (132) and abnormal microbiota (133). In fact, human gut microbiota (HGM) feeds on normal MUC2 glycans (134–136). Hence, the disruption of MUC2 glycosylation due to C1GALT1C1 mutation could potentially lead to microbiota and host physiology issue (137).

### Challenges with an ontological representation of glycophenotypes

A robust and comprehensive glycophenotype ontological representation would (i) provide synonyms of glycans between disciplines (e.g. Tn antigen or O linked *N*-acetylgalactosaminyl epitope or O-GalNAc (114)); (ii) gather identifiers (GlyTouCan, Kegg, CHEBI, JCGGDB, IUPAC, etc.); (iii) describe glycophenotypes of genetic disease with higher granularity (e.g. increase of fucosylated glycans in the urine for *ERCC6* (81)); (iv) allow comparisons between known and unknown diseases (e.g. answering questions such as ‘what diseases show an increase of fucosylated glycans in the urine?’); and (v) provide phenotype terms for annotations for biocurators. This integration will allow semantic similarity approaches for disease diagnosis based on phenotypes, including glycophenotypes, variant prioritization, patient matchmaking and model system discovery.

However, integrating glycophenotypes in an ontology creates some conceptual challenges that will require community discussion:

- #1 Determination of equivalence between native and experimental glycans is challenging and will require the harmonization of nomenclature between IUPAC, CHEBI, GlyTouCan, etc.
- #2 Alignment of logical definitions across OBO Foundry ontologies will be difficult due to the different glycobiology modeling that is represented in different contexts, and due to gaps. For example, CHEBI does not include protein; therefore, there is no place for glycoproteins; the protein ontology does not provide information about the glycan portion of a glycoprotein other than the highest level (e.g. N or O glycosylated); the GO represents only biological processes and some glycobiology processes are unknown for some phenotypes (e.g. dysfunctional DNA repair enzyme *ERCC6* is associated with increased fucosylated glycan in the urine, yet the biological process is unknown (81)).
- #3 Some phenotypes are quantitative and would require conversion to semantic qualitative descriptors, for example glycan array data where there are plots of relative fluorescence unit based on the binding of a protein to an array of hundreds of glycans.
- #4 Full granularity of the description of glycophenotypes could be challenging to navigate for curators, for example increase of fucosylation glycans versus the full name of the glycans (e.g. Neu5Ac(α2-3)Gal(β 1-4)GlcNAc(β1-3)Gal(β 1-4)[Fuc(α1-3)]GlcNAc).

The Minimum Requirement for A Glycomics Experiment, Glycomics at Expasy and GlyTouCan have joined effort to address challenge #1 with an automatic attribution of glycan identifiers from the mass spectrometry experiments (115). Nevertheless, a unification with CHEBI will be necessary (challenge #2). Indeed, glycan structures analyzed by mass spectrometry can be ambiguous; for instance, an exact hexose name and linkages can remain undetermined because of the technological limitations. While GlyTouCan tolerates this ambiguity, it differs from CHEBI’s standards. For instance, a urinary trisaccharide, assumed to be three units of glucose, can be a marker for Pompe disease (36). In this case, contrary to CHEBI, GlyTouCan can provide an identifier (G63977XF) regardless of undetermined linkage between the monosaccharides. This will necessarily reveal the gaps and lack of logical interoperability across OBO ontologies (challenge #2), but by working with each of these communities, we will be able to improve glycobiology for all contexts. Close collaboration between glycobiologists and glycoinformaticists will be required to address challenges #3 and #4.

## Towards a Glyco-Enriched Knowledge Graph of Disease for Diagnostics and Discovery

A rich set of glycophenotypes will support the integration of disease, pathway, gene function and numerous other biological knowledge (Figure 5). We have begun this integration work within the context of the Monarch knowledge graph (Figure 4) with HPO terms that are semantically associated with Mondo diseases, genes, GO terms, etc. Additionally, we are performing a broader characterization and review of glycophenotypes from the literature: for typical CDG (34), glycan-associated diseases (for instance, disease related to GBP such as Mannose-Binding lectin deficiency), genetic diseases where glycans are markers (e.g. *ERCC6* (81)) and across a spectrum of animal models (mouse, zebrafish, rat, fly, etc.). We are focusing on glycan-related knowledge, such as glycans and derivatives, GBP, glycobiology-related genes and diseases. We are also collaborating with GlyGen (https://www.GlyGen.org/) whose aim is to gather glycobiology-related data from multiple resources to provide data mining, sharing and dissemination of glycan-related information, and our curation of glycophenotypes and the Monarch knowledge graph will be made available to the GlyGen platform.

Thus far, we have limited our work on glycan representation and integration to human genetic diseases. We plan to extend this work to include data from bacterial and viral glycans and lectins in the context of infectious diseases, as attachment happens through glycans (116). For instance, Norovirus and Parvovirus lectins bind, respectively, to the glycans of ABO and P blood group antigens in host glycans, and also, host glycans and GBPs proteins can bind to pathogens (e.g. *Neisseria gonorrhoeae* (117) and *Neisseria meningitidis* (118)); therefore, human genetic variants of glycosyltransferases and lectins may play a role in microbial/viral infection (119). We believe that semantic integration at the molecular level as illustrated in Figure 5 (which shows a graph representation of the potential impact of the dysfunctional gene *C1GALT1C1* or *COSMC*) will support mechanistic discovery and identify interventional targets. A broader integration of glycophenotypes would, therefore, be a valuable part of pathways analysis tools such as Impala (120), Reactome (107) and STITCH (Search Tool for Interacting Chemicals) (121), in support of interconnecting microbiome metabolites. Finally, molecular phenotyping with glycophenotypes and pathway integration could provide better insights toward possible treatments, for instance, dietary supplementation of glycans or glycan-related molecules (122).

Ontology could be a way to integrate glycophenotypes for disease diagnosis; however, another possible approach is to integrate glycomics data to whole genome/exome sequencing (WES/WGS) as recently done by Ashikov *et al.* (123), similarly to the metabolomics integration in genomics (89, 124, 125). For a more systematic approach, a new bioinformatics pipeline integrating deep molecular glycophenotyping in WES/WGS will be needed.

In conclusion, we have discussed the importance of glycans in health and disease, the technological and informatics challenges for glycan data integration for disease discovery research and diagnostics. We have defined the concept of abnormal glycophenotypes, demonstrated their usefulness in disease diagnostic pipelines with the example of fucosidosis, proposed an integration of selected ontologies/glycoscience KBs and introduced an ontology for glycophenotypes (MGPO). Finally, we urge the community to participate in the advancement of glycophenotype representation and its inclusion in disease research KBs and in clinical diagnostic settings. For instance, glycobiologists should request new abnormal glycophenotypes terms in HPO following the guidelines (126). Similarly, clinicians should report them using the SNFG (91) and GlyTouCan (93) standards. Community coordination and knowledge integration will be critical to overcome the current knowledge gap defined herein.

## Glossary

CAZY: carbohydrate-active enzymes
CDG: congenital disorders of glycosylation
*C1GALT1C1*: core 1 synthase, glycoprotein-N-acetylgalactosamine 3-beta-galactosyltransferase 1 specific chaperone 1
CFG: consortium for functional glycomics
CHEBI: chemical entities of biological interest
ClinGen: central resource that defines the clinical relevance of genes and variants
ClinVar: public archive of interpretations of clinically relevant variants.
DB: database
*ERCC6*: excision repair 6, chromatin remodeling factor
Fuc: fucose
Gal: galactose
GalNAc: N-acetylgalactosamine
GBP: glycan-binding protein (lectin)
Glc: glucose
GlcNAc: *N*-acetylglucosamine
GlyConnect: platform for glycoscience data
GlycoSciences: KB for glycoscience data
GlyTouCan: repository for glycans
GO: gene ontology
HGNC: Human Genome Organisation Gene Nomenclature Committee
HMO: human milk oligosaccharides
HPO: human phenotype ontology
HS: heparan sulfate
ID: identifier
Ig: immunoglobulin
IUPAC: International Union of Pure and Applied Chemistry
JCGGDB: Japan Consortium for Glycobiology and Glycotechnology DataBase
KB: knowledge base
KEGG: Kyoto Encyclopedia of Genes and Genomes
LOINC: logical observation identifiers names and codes
LPS: lipopolysaccharide
MGPO: Molecular GlycoPhenotype Ontology
Mondo: Monarch Disease Ontology
MP: Mammalian Phenotype Ontology
NCIt: National Center Institute thesaurus
Neu5Ac: N-acetylneuraminic acid
NIH: the National Institutes of Health
OBO: open biological and biomedical ontology
OMIM: Online Mendelian Inheritance in Man
Orphanet: KB on rare diseases
PRO: protein ontology
Pubchem: NIH’s chemistry KB
Reactome: pathway KB
SNFG: symbol nomenclature for glycans
SNOMED CT: Systematized Nomenclature of Medicine Clinical Terms
STITCH: search tool for interacting chemicals
UDP: Undiagnosed Diseases Program
UDN: Undiagnosed Diseases Network
UniLectin: KB for glycan-binding protein
uPheno: unified phenotype ontology
WES: whole exome sequencing
WGS: whole genome sequencing

## Supplementary Material

Supplemental-Table1_baz114

Supplemental-Table2_baz114
